# Association Between Amyloid Accumulation and Sleep in Patients With Idiopathic REM Sleep Behavior Disorder

**DOI:** 10.3389/fneur.2020.547288

**Published:** 2020-12-03

**Authors:** Hanul Lee, Hyunjin Cho, Yeong Sim Choe, Sang Won Seo, Eun Yeon Joo

**Affiliations:** ^1^Department of Neurology, Neuroscience Center, School of Medicine, Samsung Medical Center, Sungkyunkwan University, Seoul, South Korea; ^2^Department of Health Sciences and Technology, Samsung Advanced Institute for Health Sciences & Technology (SAIHST), Sungkyunkwan University, Seoul, South Korea

**Keywords:** idiopathic REM sleep behavior disorder, amyloid, sleep, cognition, default mode network

## Abstract

**Background and Objectives:** Amyloid-beta protein may lead to sleep disturbance and eventually develop cognitive impairment. Idiopathic rapid eye movement (REM) sleep behavior disorder (iRBD) is a predictor of neurodegeneration, yet there have been limited studies evaluating the relationship between cognitive decline and amyloid accumulation in iRBD patients. The aim of this study is to investigate the clinical and sleep characteristics of iRBD patients and its association with amyloid deposition.

**Methods:** We enroll 23 iRBD patients (mean age, 65.8 years; male, 73.9%), and their mean history of clinically suspected RBD was 6.5 years. All underwent 18F-flutemetamol amyloid PET completed polysomnography (PSG) and questionnaires. Patients were classified into two groups according to amyloid deposition as amyloid positive and negative. Clinical and sleep parameters were compared between groups and were correlated with amyloid deposition, calculated as a standardized uptake value ratio (SUVR).

**Results:** Four patients (17.4%) were revealed to be amyloid positive, and they showed increased percentage of wake after sleep onset (WASO), stage N1, and stage N2 sleep and worse on the Stroop Word Color Test compared to amyloid negative patients. Global SUVR was correlated with total sleep time, sleep efficiency, WASO, and N1 sleep, and these sleep parameters were associated with a part of default mode network of brains such as orbitofrontal, dorsolateral pre-frontal, and left temporal areas.

**Conclusion:** iRBD patients with amyloid deposition have worse sleep quality than patients without amyloid. Relationship between fragmented sleep and amyloid deposition in the default mode network may be crucial to elucidate the disease progress of iRBD.

## Introduction

Rapid eye movement (REM) sleep behavior disorder (RBD) is a well-known parasomnia characterized by absence of muscle atonia during REM sleep. RBD presents with dream enacting motor behaviors or vocalization during REM sleep, such as sleep talking, screaming, punching, or falling off the bed (1). These behaviors are associated with violent contents of the dream during REM sleep (2), which may eventually result in physical injury to patients or bedside partners.

The term idiopathic RBD (iRBD) is used in the absence of other known causes for secondary RBD, which are mainly neurodegenerative diseases associated with synucleinopathy, such as dementia with Lewy bodies, Parkinson's disease, or multiple system atrophy (3, 4). The association of iRBD with synucleinopathy is well-established (5, 6). Evidence has increased regarding phenoconversion of iRBD into synucleinopathies presenting with parkinsonism or dementia (5, 7, 8). The relationship between iRBD and synucleinopathy corresponds with the progression of disease pathology affecting brain anatomical structures (9, 10).

Numerous data showed that iRBD patients perform poorly on neuropsychological tests (11–16), and mild cognitive impairment (MCI) is frequently observed in iRBD patients (14, 17, 18). However, results were inconsistent across studies depending on which cognitive domain was impaired, population heterogeneity, small sample size, and the use of different cognitive tasks with variable sensitivity (19).

Amyloid-beta protein is known as one of the key molecules in pathophysiology of Alzheimer's disease (AD), which is the most common cause of dementia in the elderly and characterized by memory deficit and cognitive dysfunction (20). The primary cause for the disease is not completely known; thus, mixed proteinopathy of amyloid and tau is the proposed pathology and results in amyloid plaque and neurofibrillary tangle deposition (21). Amyloid beta may be increased with the normal aging process and show positivity among the cognitively normal elderly. The deposition of amyloid would result in cognitive decline in episodic memory, visuospatial function, and global cognition (22). Recent neuroimaging techniques such as amyloid positron emission tomography (PET) represents a potential major advance in the assessment of subjects with cognitive impairment by binding fluorescent isotopes in fibrillary beta amyloid in the brain tissues (23).

Amyloid deposition, sleep, and cognitive function are closely interrelated. AD patients show substantial alteration in sleep structure and quality due to amyloid deposition (24). This change develops during the early stage of disease before the cognitive symptom appears (25). Furthermore, the sleep disturbance increases cerebral amyloid beta level and subsequently elevates the risk of AD (26). RBD symptoms are not uncommon in AD patients (27, 28), and cognitive decline is commonly observed in iRBD patients (11–16). The relationship between amyloid deposition and RBD symptoms and the role of brain amyloid in the disease course of iRBD have not been clarified yet. In the present study, we hypothesize that amyloid accumulation contributes to sleep disturbance and eventually cognitive impairment in iRBD patients and aims to investigate its relationships with sleep and cognition in those patients.

## Materials and Methods

### Study Participants

A total of 32 treatment-naïve iRBD patients who consecutively visited the Samsung Medical Center sleep clinic between June 2017 and January 2019 were enrolled in the present study. The diagnosis of iRBD is based on the International Classification of Sleep Disorders 3rd edition (ICSD-3) criteria (29).

All participants provided written informed consent and the study protocol was approved by the Ethics Committee of Samsung Medical Center. Patients who reported typical RBD motor or vocalization symptoms were eligible for the study. Two individual neurologists assessed patients to determine cognitive dysfunction or parkinsonism during the initial enrollment. Five patients with a history of cancer, three with a history of minor stroke, and one with parkinsonian features were excluded. Finally, 23 patients completed questionnaires including registration survey, RBD questionnaire Korean version (RBDQ-KR), Epworth Sleepiness Scale (ESS), Beck Depression Inventory (BDI), Geriatric Depression Scale (GDS-K), Hospital Anxiety and Depression Scale (HADS), Pittsburgh Sleep Quality Index (PSQI), and the Scales for Outcomes in Parkinson's disease-Autonomic (SCOPA-AUT). Registration survey contained questions about alcohol, caffeine consumption, habitual sleep time, habitual sleep latency, underlying disease, and onset of RBD symptom. In particular, alcohol consumption was assessed using questions about average weekly consumption during the past 12 months (30). All participants fulfilled sleep diary more than 2 weeks before enrollment. Sleep latency and sleep duration were calculated from the data.

Patients' cognitive function and parkinsonism were evaluated using the Korean version of the Mini-Mental State Examination (K-MMSE), Clinical Dementia Rating scale (CDR), Korean version of Montreal Cognitive Assessment (K-MOCA), and Unified Parkinson's Disease Rating Scale (MDS-UPDRS). After enrollment, all patients underwent overnight polysomnography (PSG), neuropsychological test, brain MRI, and 18F-flutemetamol PET scan.

### Sleep Study (Overnight Polysomnography)

Polysomnography (PSG) was recorded with standard electrodes and sensors using Embla N7000 (Medcare Flaga, Iceland). The study was performed using the standard PSG protocol with C3–A2, C4–A1, F3–A2, F4–A1, O3–A2, and O2–A1 electroencephalography electrodes, four electrooculography electrodes, chin, and both anterior tibialis electromyogram, and electrocardiography sensors were monitored. Thoracic and abdominal movement were monitored using two plethysmography belts, nasal and oral airflow was measured using a nasal pressure transducer, and oxygen saturation was monitored using a thermistor and pulse oximetry. Synchronized video recording was used to monitor abnormal sleep behaviors or vocalization during sleep. Sleep parameters such as total sleep time (TST), sleep latency, waking after sleep onset (WASO), sleep efficiency (SE), sleep stages (N1, N2, N3, REM sleep, %), arousals (arousal index, respiratory-related arousal, spontaneous arousal, movement-related arousal), and the apnea–hypopnea index (AHI) were collected. Sleep stages and sleep events scoring were defined according to American Academy of Sleep Medicine (AASM) manual for sleep and associated events (31).

### Neuropsychological Test

All patients completed a standardized neuropsychological battery, the Seoul Neuropsychological Screening Battery (SNSB), to evaluate cognitive function (32). One neuropsychological specialist had done every patient's test. Test was done by face-to-face examination, including interviewing the patient's caregivers. The battery is composed of multiple tests to assess attention and executive function domains such as information processing, verbal fluency, verbal memory, visual memory, and language. The score of the individual test was computed as *z*-score and averaged as composite score for each domain.

### Brain MRI Scanning

Participants underwent a 3D volumetric brain MRI scan. An Achieva 3.0 T MRI scanner (Philips; Best, the Netherlands) was used to acquire 3D T1 turbo field echo MRI data. Imaging parameters were as follows: sagittal slice thickness of 1.0 mm with 50% overlap, no gap, repetition time of 9.9 ms, echo time of 4.6 ms, flip angle of 8°, and matrix size of 240 × 240 pixels reconstructed to 480 × 480 pixels over a 240-mm field of view.

### 18F-Flutemetamol Amyloid PET Scanning

Flutemetamol PET scans were conducted with a Discovery 600 PET/CT scanner (GE), Discovery 690 PET/CT scanner (GE), Discovery STE PET/CT scanner (GE), Biography MCT PET/CT scanner (Siemens), or Gemini TF PET/CT scanner (Philips) (33). The participants underwent a 20-min PET scan (4 × 5 min dynamic frames) starting at 90 min after intravenous injection of 185 MBq ± 10% of 18F-flutemetamol. Low-dose CT was utilized for attenuation correction before scans. Visual interpretation of 18F-flutemetamol PET images relied upon a systematic review of five brain regions (frontal, parietal, posterior cingulate and precuneus, striatum, and lateral temporal lobes). If any of the brain regions systematically reviewed for 18F-flutemetamol PET was positive in either hemisphere, the scan was considered positive.

Flutemetamol PET images were co-registered to individual MRIs, which were normalized to a T1-weighted MRI template. The quantitative regional values of flutemetamol retention on the spatially normalized flutemetamol images were obtained using an automated volume of interest (VOI) analysis with the automated anatomical labeling atlas. Data processing was performed using the Statistical Parametric Mapping (SPM) program, Version 8 in the Matlab 2014b (Mathworks, Natick, MA, United States). A total of 28 cortical VOIs were selected from each hemisphere using the AAL atlas. Regional cerebral standardized uptake value ratios (SUVRs) of flutemetamol were calculated by dividing each cortical VOI's SUV by the mean SUV of the pons value (a reference). The cerebral cortical VOIs chosen for this study consisted of frontal areas (superior and middle frontal gyri, the medial part of the superior frontal gyri, the opercular and triangular parts of the inferior frontal gyri, the supplementary motor areas, the orbital part of the superior, middle, and inferior orbital frontal gyri, the rectal gyri, and the olfactory cortices), posterior cingulate gyri, parietal areas (superior and inferior parietal areas, supramarginal and angular gyri, and precuneus), lateral temporal areas (superior, middle, and inferior temporal gyri and Heschl's gyri), and occipital areas (superior, middle, and inferior occipital gyri, cuneus, calcarine fissures, and lingual and fusiform gyri) bilaterally. A global flutemetamol uptake was calculated from the volume-weighted average SUVR of 28 cortical VOIs from each hemisphere.

### Voxel-Wise Regression Analysis

SPM analysis was performed without global count normalization because the flutemetamol PET images were changed to the uptake ratio (SUVR) parametric image using the pons region of interest (ROI) uptake value. Statistical comparisons between sleep parameters and SUVR were performed on a voxel-by-voxel basis using linear regression with age as covariate. Statistically increased flutemetamol retention areas of the brain were investigated at a height threshold of false discovery rate-corrected *p* < 0.05 with an extent threshold of 100 voxels. The MNI coordinates of the local maximum of each cluster were converted into Talairach coordinates.

### Statistical Analysis

All continuous variables were analyzed using the independent *t*-test or Mann–Whitney *U*-test, and categorical variables were analyzed using Fisher's exact test. Correlation between clinical and PSG parameters and PET SUVR values were analyzed using Pearson correlation test or Spearman correlation. Age and education years were used as control variables for correlation analysis. Statistical analyses were performed with SPSS software (version 25.0, SPSS Inc., Chicago, IL, United States). A *p* < 0.05 was considered to indicate statistical significance.

## Results

Out of 23 iRBD patients who completed full evaluations, 16 patients (69.5%) were confirmed by typical RBD behaviors correlated with simultaneously occurring dream mentation as well as REM without atonia (RWA) on polysomnographic recording. The remaining seven patients demonstrated sufficient RWA although RBD behaviors did not exhibit on sleep study night. All patients had typical clinical history of RBD with dream-enacting behaviors. Clinically suspected RBD history was varied from 2 to 10 years (mean 6.5 years).

Four patients (17.4%) were positive on 18F-flutemetamol PET. Demographics and clinical data were not different between amyloid-positive and amyloid-negative patients (Table 1).

**Table 1:** Demographics and clinical information in patients with idiopathic RBD.

	**Total**	**Amyloid (+)**	**Amyloid (–)**	***p*-value**
Age	65.80 (60.30–69.80)	70.60 (59.18–74.53)	65.50 (60.30–68.60)	0.194
Disease onset age	59.00 (50.00–66.00)	63.00 (52.25–73.00)	57.00 (50.00–65.00)	0.330
Sex, Male:Female	17:6	4:0	13:6	0.539
Body mass index, kg/m^2^	24.90 (23.40–26.70)	25.70 (24.72–28.17)	24.90 (23.25–26.40)	0.282
Hypertension	8	0	8	0.257
Type II diabetes	2	0	2	1.000
Alcohol, *n*/week	0.00 (0.00–1.00)	0.13 (0.00–0.44)	0.00 (0.00–1.00)	0.653
Caffeine, *n*/day	1.00 (0.00–2.50)	0.00 (0.00–1.88)	1.00 (1.00–3.00)	0.081
Habitual sleep time, h	6.50 (5.75–7.63)	7.00 (5.00–7.88)	6.50 (5.75–7.25)	0.731
Habitual sleep latency, h	0.28 (0.10–0.50)	0.50 (0.20–0.88)	0.25 (0.10–0.50)	0.161
Epworth sleepiness scale	5.00 (3.25–8.75)	6.00 (3.25–9.50)	5.00 (3.25–8.75)	0.887
Pittsburgh sleep quality index	3.00 (2.00–4.00)	4.00 (3.00–4.00)	3.00 (2.00–3.25)	0.147
Insomnia severity index	9.00 (4.00–14.50)	5.00 (3.00–14.00)	9.50 (3.75–15.25)	0.569
Stanford sleepiness scale	2.00 (2.00–3.00)	2.00 (1.25–3.50)	2.50 (2.00–3.00)	0.548
RBDQ-KR	39.00 (29.00–63.50)	42.00 (37.00–50.00)	37.00 (27.50–64.25)	0.887
K-MMSE	28.00 (25.00–30.00)	26.50 (21.25–29.50)	29.00 (27.00–30.00)	0.299
Clinical dementia rating scale	0.50 (0.00–0.50)	0.50 (0.12–0.50)	0.00 (0.00–0.50)	0.325
Beck depression inventory II	12.00 (9.00–17.00)	7.50 (5.00–7.88)	16.00 (9.00–20.00)	0.056

*RBDQ-KR, REM sleep behavior disorder Questionnaire-Korean version; K-MMSE, Korean version of Mini-Mental Statue Examination. All variables are presented as median and interquartile range (Q1–Q3)*.

* *p < 0.05, Mann–Whitney U-test*.

In sleep data, the amyloid-positive group had significantly higher N1 sleep time (*p* = 0.014) and lower N2 sleep time (*p* = 0.027) than the amyloid-negative group (Table 2).

**Table 2:** Comparison of polysomnography parameters in in patients with idiopathic RBD according to amyloid deposition.

	**Amyloid (+)**	**Amyloid (–)**	***p*-value**
Time in bed, min	455.30 (375.90–516.63)	461.00 (403.75–479.00)	0.929
Total sleep time, min	256.25 (204.25–399.00)	384.50 (320.13–438.50)	0.148
Sleep latency, min	9.00 (3.25–11.38)	7.00 (4.25–17.13)	0.733
REM sleep latency, min	80.25 (64.50–166.50)	74.50 (57.13–96.38)	0.610
Sleep efficiency, %	67.35 (42.70–83.23)	89.00 (71.70–91.40)	0.074
N1 sleep time, %	31.60 (18.60–41.08)	15.45 (8.00–21.13)	0.014^*^
N2 sleep time, %	51.05 (42.80–55.93)	63.30 (54.53–67.53)	0.027^*^
N3 sleep time, %	0.00 (0.00–0.00)	0.25 (0.00–1.38)	0.063
REM sleep time, %	17.35 (4.88–36.73)	22.90 (14.83–26.63)	0.551
WASO, min	133.30 (59.00–272.40)	43.40 (33.90–100.50)	0.052
WASO, %	31.10 (14.68–56.23)	10.00 (7.70–25.70)	0.052
Apnea-hypopnea index, /h	9.40 (2.68–15.83)	9.00 (1.90–17.00)	0.935
Arousal index, /h	23.60 (16.30–24.53)	14.45 (8.65–27.35)	0.733
Respiratory arousal	5.40 (2.05–6.28)	5.50 (1.05–11.45)	0.858
Snore arousal	0.60 (0.33–6.35)	0.20 (0.00–0.65)	0.073
RERA	4.40 (1.78–7.70)	1.60 (0.02–2.75)	0.065
Spontaneous arousal	7.90 (6.33–9.33)	5.15 (2.28–9.65)	0.394
Movement related arousal	0.95 (0.15–3.85)	0.10 (0.00–1.20)	0.434
PLM index, h	13.05 (1.25–48.33)	5.40 (0.00–28.30)	0.709

*REM, Rapid eye movement; WASO, Wake after sleep onset; RERA, Respiratory effort related arousal; PLM, Periodic limb movement during sleep. All variables are presented as median and interquartile range (Q1–Q3)*.

* *p < 0.05, Mann–Whitney U-test*.

Amyloid-positive patients showed worse performance in the Stroop Word Test (*p* = 0.003) and Stroop Color Test (*p* = 0.015; Table 3). Seven of 23 patients (30.4%) were revealed to have MCI state, and two of them were amyloid-positive. The numbers of MCI were not different between amyloid-positive and amyloid-negative groups (50 vs. 35.71%, Fisher's exact test, *p* = 0.557).

**Table 3:** Comparison of neuropsychological data in patients with idiopathic RBD according to amyloid deposition.

	**Amyloid (+)**	**Amyloid (–)**	***p*-value**
Frontal and executive function composite score	−0.900 (−1.483–0.615)	0.126 (−0.342–0.564)	0.138
Digit symbol test	37.00 (34.00–75.25)	67.00 (44.00–83.00)	0.223
Controlled oral word association test, phonemic	15.50 (8.25–40.00)	24.00 (19.00–36.00)	0.256
Controlled oral word association test, semantic	31.00 (17.00–38.25)	37.00 (26.00–50.00)	0.239
Train making test A, time to completion	30.00 (11.75–49.75)	20.00 (16.00–30.00)	0.350
Train making test B, time to completion	71.00 (20.75–164.00)	34.00 (26.00–71.00)	0.464
Stroop tests word, correct responses	109.50 (103.00–111.50)	112.00 (112.00–112.00)	0.003^*^
Stroop tests color, correct responses	65.50 (52.75–85.75)	109.00 (83.00–112.00)	0.015^*^
Attention composite score	−0.484 (−1.036–1.010)	0.135 (−0.484–1.082)	0.456
Digit span, forward	6.00 (5.25–8.25)	7.00 (6.00–8.00)	0.506
Digit span, backward	4.00 (3.00–6.50)	5.00 (4.00–6.00)	0.561
Verbal memory composite score	−0.880 (−2.000–0.405)	0.005 (−0.383–0.894)	0.081
Seoul verbal learning test, total	19.50 (12.25–24.50)	22.00 (18.00–26.00)	0.290
Seoul verbal learning test, delayed response	2.00 (0.00–7.00)	7.00 (5.00–10.00)	0.054
Seoul verbal learning test, recognition	20.00 (17.00–23.00)	22.00 (21.00–23.00)	0.169
Visual memory composite score	−0.499 (−1.376–0.393)	0.037 (−0.403–0.675)	0.188
Rey complex figure test, copy	31.50 (28.00–33.50)	34.00 (30.00–36.00)	0.265
Rey complex figure test, immediate	17.25 (6.38–21.00)	21.50 (16.00–24.50)	0.180
Rey complex figure test, delayed	12.50 (4.25–22.25)	18.50 (14.00–24.00)	0.133
Rey complex figure test, recognition	20.00 (18.50–20.75)	20.00 (19.00–21.00)	0.834
Language			
Korean-boston naming test	47.50 (38.25–53.00)	51.00 (48.00–55.00)	0.409

*All variables are presented as median and interquartile range (Q1–Q3)*.

* *p < 0.05, Mann–Whitney U-test*.

**Figure 1:**
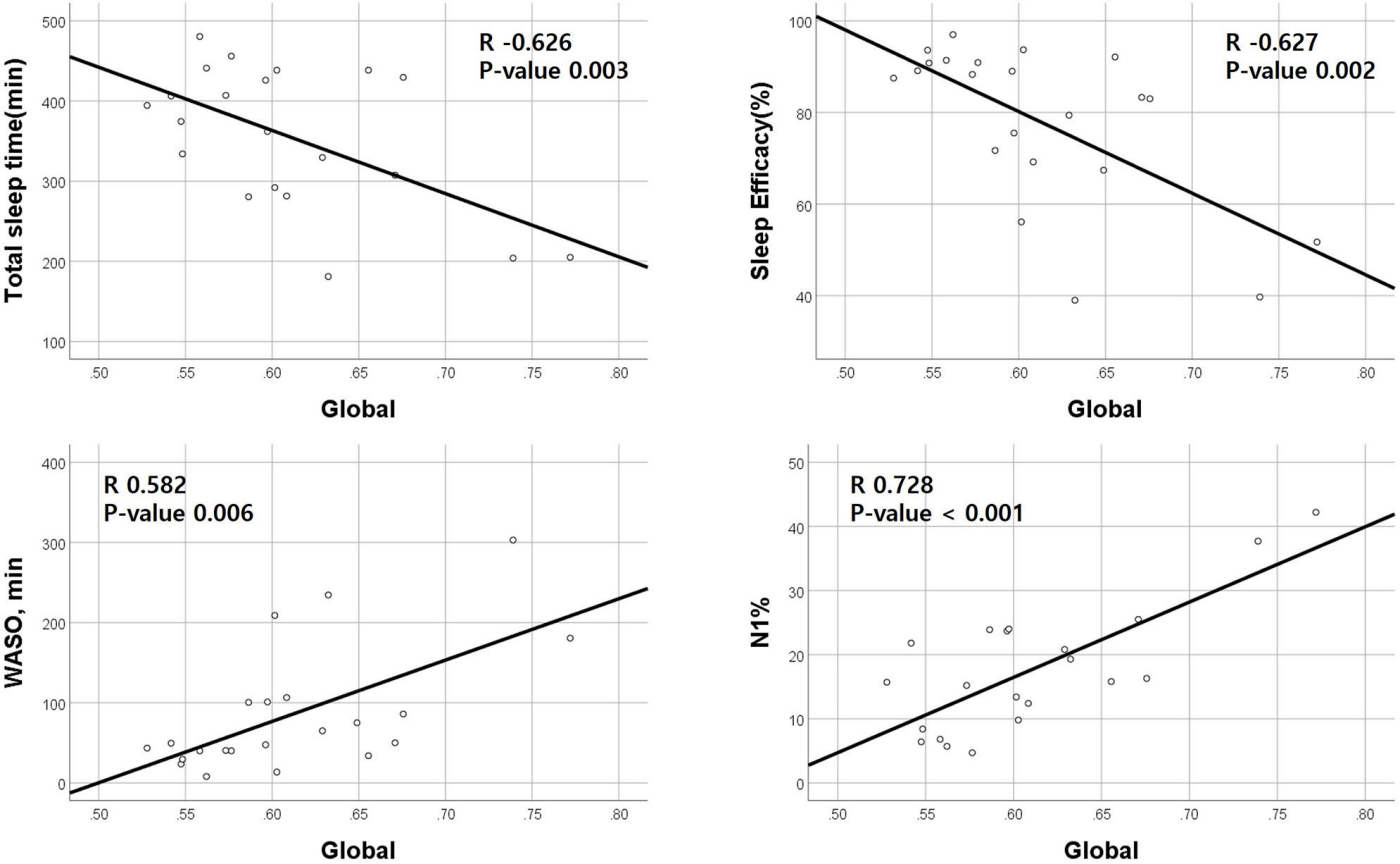
Correlations between positron emission tomography standardized uptake value ratio (PET SUVR) and polysomnography parameters. Amyloid deposition (SUVR) is negatively correlated with total sleep time and sleep efficiency and positively correlated with wakefulness after sleep parameters (WASO) and N1 sleep %. The *X*-axis represents the amount of global amyloid deposition.

Correlation analyses between clinical data and imaging data were performed in 23 patients. A global amyloid burden based on SUVR was negatively correlated with TST (Pearson's coefficient −0.626, *p* = 0.003) and SE (−0.627, *p* = 0.002) and positively correlated with WASO (0.582, *p* = 0.006) and N1 sleep time (0.728, *p* < 0.001; Figure 1). SVLT total score was also negatively correlated with RBDQ-KR (−0.511, *p* = 0.025). RBDQ-KR showed a tendency to have negative correlation with verbal memory composite score (*p* = 0.064).

**Figure 2:**
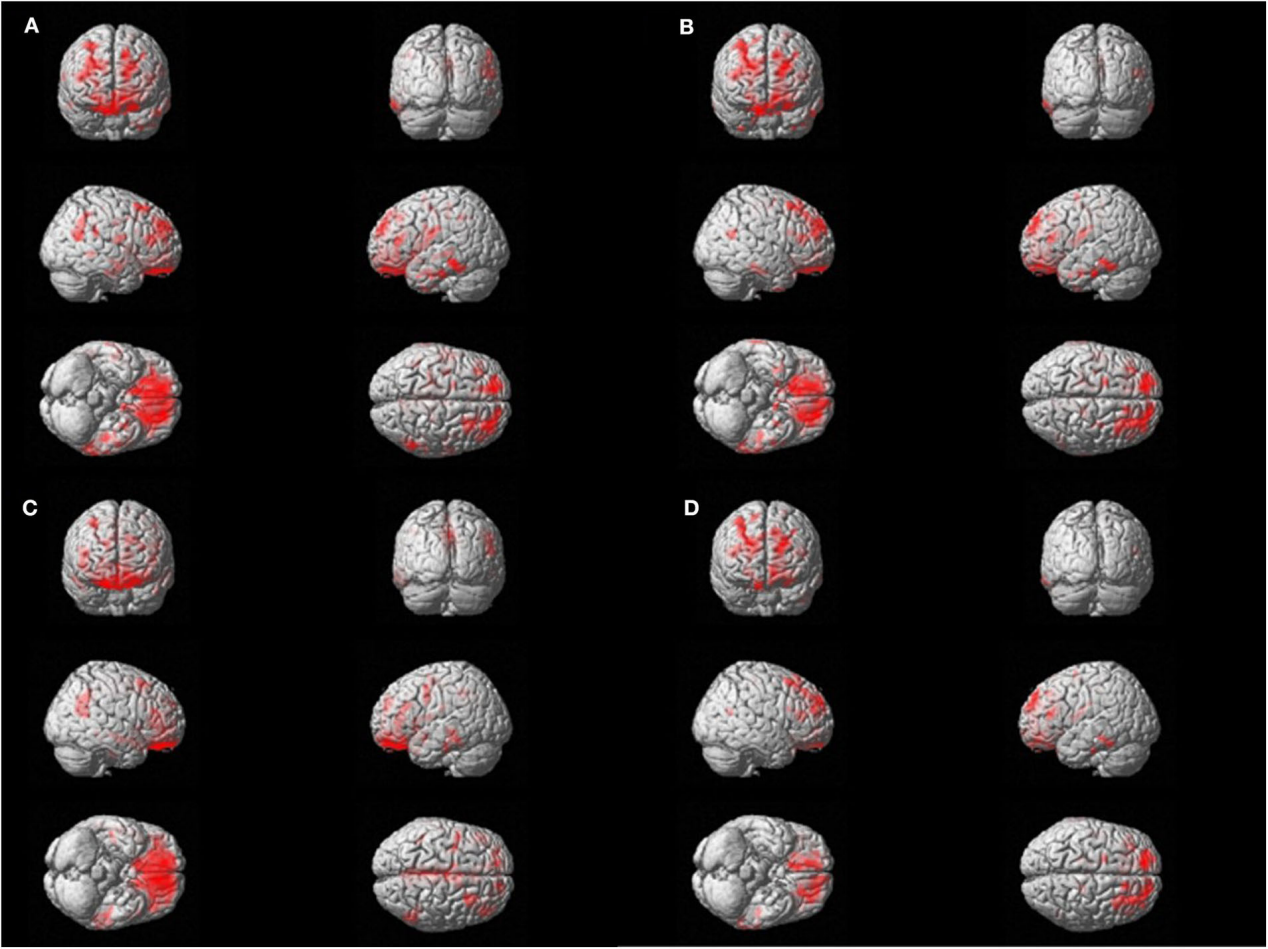
Voxel-wise regression analysis between amyloid uptake and polysomnography parameters. Significant correlations are found with amyloid positron emission tomography standardized uptake value ratio (PET SUVR) and the sleep parameters in bilateral orbitofrontal, dorsolateral prefrontal, and left lateral temporal areas. The red area represents the three-dimensional location of areas that has correlation with each sleep parameter. FDR correction was done (*p* < 0.05). **(A)** Total Sleep Time, min. **(B)** Sleep efficiency, %. **(C)** Wakefulness after sleep onset, min. **(D)** N1 time, %.

Voxel-wise regression analysis was performed with four parameters (TST, SE, WASO, and N1 sleep time), which were significant in the global SUVR correlation analyses and showed relevant associations with cortical retention of flutemetamol in bilateral orbitofrontal, dorsolateral pre-frontal, and left lateral temporal areas (*p* < 0.05, False discovery rate corrected; Figure 2).

## Discussion

In the present study, amyloid deposit was hypothetically responsible for sleep disturbance and cognitive impairment in iRBD patients and the relationships between amyloid burden and sleep and cognitive data of patients were investigated. Four patients (17.4%) were amyloid-positive on PET scan and their sleep quality was worse than in amyloid-negative patients. Several cognitive domains were more impaired in amyloid-positive patients, but the percentage of patients with MCI state was not higher than amyloid-negative patients.

Considering the median age of study participants (63.3 years), amyloid-positive rate (17.4%) in iRBD patients was comparable to that of cognitively normal elderly in a population-based study (18.3%) (34). Literature showed that RBD symptoms were manifested in 10% of AD patients (27, 28) and in 1.06% of the cognitively normal elderly population (35). RBD is regarded as the prodromal feature of synucleidopathy diseases (5, 7, 8, 27, 36, 37), whereas it is accompanied with AD patients in relatively small portion (10%) (27, 28). Cognitive decline is commonly observed in iRBD patients and prevalence of MCI in RBD patient is up to 35–50% (14, 38). This is the first study to investigate the positivity rate of amyloidopathy in iRBD patients and the association between RBD and other neurodegenerative diseases.

iRBD patients in this study also reported overall good sleep quality (PSQI and ISI) and daytime function (ESS and SSS), which in line with previous reports (1, 39). Subjective cognitive function was also well-preserved (K-MMSE and CDRS). In spite of the fact that amyloid-group differences of the above parameters were not significant, objective sleep parameters of amyloid-positive patients presented more fragmented sleep than amyloid-negative patients. Amyloid-positive iRBD patients had smaller N1 and larger N2 sleep %. Sleep efficiency was much decreased in the amyloid-positive group (64.43%, normal ≥ 85%), but statistically indifferent with amyloid negative patients (81.6%, *p* = 0.074). WASO % also showed a tendency to be increased in amyloid-positive patients (34.00% in amyloid positive vs. 16.76% in amyloid negative, *p* = 0.052). The small number of amyloid-positive patients (*n* = 4) may limit the statistical significance. From the clinical point of view, however, reduced sleep efficiency and increased WASO (more than 1/3 of total sleep) of amyloid-positive iRBD patients were enough to be interpreted as poor, fragmented sleep.

Intriguingly, sleep latency and AHI were within normal range considering ages in both groups and arousal index and periodic limb movement index were increased. It indicates that amyloid-positive iRBD patients have more fragmented sleep and worse sleep quality regardless of sleep-related breathing disorder, periodic limb movement disorder (PLMD), or sleep onset insomnia, and it enlightens the role of brain amyloid in development of sleep disturbances independent of specific sleep disorders in iRBD patients. With cognitive decline, the sleep becomes worse and shorter REM sleep and N3 sleep time and reduced sleep efficiency were found in AD patients (40). Taken together with our data, it is presumable that amyloid deposition in the brain may worsen the sleep efficiency in iRBD patients as well as cognitive-normal people (25).

Regarding neuropsychological testing, several frontal and executive function domains were worse in amyloid-positive patients. Four patients were revealed to be MCI state but dementia was not found. MCI state rate was not different according to amyloid deposition. More severe RBD symptoms scored by RBDQ-KR were significantly associated with impaired memory in iRBD patients. Previous reports showed that RBD patients had cognitive impairment in specific domains, such as attention, executive function, verbal and non-verbal memory, and visuospatial abilities (11–15). In particular, the Stroop Color Word Test and Trail Making Test are known to predict the development of dementia in iRBD patients (41). Participants in this study did not report subjective cognitive impairment. Notably, amyloid-negative patients did not show lower scores in any neuropsychological domains than amyloid-positive patients. MCI occurs in 35–50% of iRBD patients (14) and a similar rate was observed in this study (30.4% of 23). However, MCI prevalence was not different between amyloid-positive and amyloid-negative groups. It is uncertain whether amyloid burden is associated with the development of MCI based on these data.

Brain regions with high neuronal activity are considered more susceptible to amyloid deposition. The default mode network consists of precuneus, posterior cingulate, medial pre-frontal cortex, lateral temporal cortex, hippocampal formation, angular gyrus, and retrosplenial cortex, and it is regarded as the most vulnerable region to amyloid burden (42). A previous report in non-demented late middle-aged adults showed that sleep disturbances estimated using the ESS and Medical Outcomes Study Sleep Scale were associated with amyloid burden in default mode network (angular gyrus, frontal medial orbital cortex, cingulate gyrus, and precuneus) (43). A survey of community-dwelling older adults found that short sleep duration was associated with amyloid deposition in the brain (44).

iRBD patients in this study report good sleep quality (PSQI) and do not complain daytime sleepiness or insomnia related to sleep disturbances. They sleep enough (average 6.48 h) and have regular sleep–wake pattern. However, a sleep study showed that patients had lower sleep efficiency and, in particular, amyloid-positive patients have more fragmented sleep. Essential parameters (TST, sleep efficiency, WASO, and N1 sleep time) for sleep integrity were significantly correlated with amyloid deposition in the part of default of network as well as global amyloid burden.

Since this is a cross-sectional study, it does not clarify the causal relationships among sleep disturbance, cognitive decline, and amyloid deposition in iRBD patients. We observed in this study that amyloid-positive patients showed more poor sleep quality and worse performance in some parts of neuropsychological tests. Two out of four amyloid-positive patients were revealed to have MCI state. Although the numbers of MCI were not different between amyloid-positive and amyloid-negative groups, it is presumable that amyloid deposition may lead to sleep disturbance and then cognitive decline in iRBD patients.

The present study had several limitations. First, the study included a small number of iRBD patients that have weakened the statistical power and overestimated the results. Second, amyloid-positive healthy controls without RBD symptoms were not included to evaluate the effects of amyloidopathy on iRBD. Third, the technical limitation of amyloid PET scan may hinder the interpretation of the results. Brainstem structures associated with RBD (45) are too small to evaluate with current PET technology (46). Despite the limitations, it has the strength that it demonstrates that the amyloid-positive iRBD patients have more disturbed sleep and more cognitive impairment compared with amyloid-negative patients for the first time and it suggests the possibility that early detection and intervention of sleep disturbance may prevent or delay the conversion to dementia in iRBD patients.

## Data Availability Statement

The raw data supporting the conclusions of this article will be made available by the authors, without undue reservation.

## Ethics Statement

The studies involving human participants were reviewed and approved by Ethics Committee of Samsung Medical Center. The patients/participants provided their written informed consent to participate in this study.

## Author Contributions

HL, SS, and EJ contributed to conception and design of the study. HL, HC, and YC organized the database. YC contributed to image processing and figure making. HL wrote the first draft of the manuscript. All authors contributed to manuscript revision and read and approved the submitted version.

## Conflict of Interest

The authors declare that the research was conducted in the absence of any commercial or financial relationships that could be construed as a potential conflict of interest.
